# The importance of targeting signalling mechanisms of the SLC39A family of zinc transporters to inhibit endocrine resistant breast cancer

**DOI:** 10.37349/etat.2022.00080

**Published:** 2022-04-26

**Authors:** Samuel Jones, Georgia Farr, Thirayost Nimmanon, Silvia Ziliotto, Julia M.W. Gee, Kathryn M. Taylor

**Affiliations:** 1 School of Pharmacy and Pharmaceutical Sciences, Cardiff University, CF10 3NB Cardiff, UK; 2 Department of Pathology, Phramongkutklao College of Medicine, Bangkok 10400, Thailand; 3 School of Biosciences, Cardiff University, CF10 3AX Cardiff, UK

**Keywords:** Zinc transport, SLC39A7, SLC39A6, SLC39A10, tamoxifen resistance, STAT3, ZIP6, ZIP7

## Abstract

**Aim:**

Zinc is a key secondary messenger that can regulate multiple signalling pathways within cancer cells, thus its levels need to be strictly controlled. The Zrt, Irt-like protein (ZIP, SLC39A) family of zinc transporters increase cytosolic zinc from either extracellular or intracellular stores. This study examines the relevance of zinc transporters ZIP7 and ZIP6 as therapeutic targets in tamoxifen resistant (TAMR) breast cancer.

**Methods:**

A series of *in vitro* assays, including immunohistochemistry, immunofluorescence, flow cytometry, and western blotting were used to evaluate levels and activity of ZIP7 and ZIP6 in models of TAMR and sensitive (MCF-7) breast cancer. Analyses of these transporters in the clinical setting were performed using publicly available online resources: Gene Expression Profiling Interactive Analysis (GEPIA)2 and Kaplan-Meier Plotter (KmPlot).

**Results:**

Both total and activated levels of ZIP7 were significantly elevated in TAMR cells *versus* responsive MCF-7 cells. This was accompanied by an associated increase in free cytoplasmic zinc leading to amplification of downstream signals. Consistent with our proposed model, activated ZIP6 levels correlated with mitotic cells, which could be efficiently inhibited through use of our anti-ZIP6 monoclonal antibody. Mitotic inhibition translated to impaired proliferation in both models, with TAMR cells displaying increased sensitivity. Analysis of matched tumour and normal breast samples from patients revealed significant increases in both ZIP7 and ZIP6 in tumours, as well as family member ZIP4. Kaplan-Meier analysis revealed that high ZIP7 levels correlated with decreased overall and relapse-free survival (RFS) of patients, including patient groups who had received systemic endocrine therapy or tamoxifen only. In contrast, high ZIP6 levels were significantly linked to improved overall and RFS in all patients, as well as RFS in patients that received systemic endocrine therapy.

**Conclusions:**

TAMR cells displayed increased activity of both ZIP7 and ZIP6 transporters compared to anti-hormone responsive cells, suggesting their potential as novel therapeutic targets following development of resistant disease.

## Introduction

It has long been recognized that zinc intake is an essential part of maintaining normal physiology, with its dysregulation being directly attributed to a number of disease states [1]. More recently, the elucidation of zinc regulation at a cellular level has revealed its capacity as a central messenger in a wide range of signalling cascades; most importantly as the key regulator of cell progression into mitosis [2].

Similar to other metallic elements, zinc is unable to passively enter cells through the plasma membrane and likewise requires the use of specific zinc transporters to regulate its homeostasis. These exist in two families: the zinc transporter protein (ZnT, SLC30A) family and the Zrt, Irt-like protein (ZIP, SLC39A) family. The 10 family members of ZnT transporters primarily shuttle zinc out of the cytoplasm of cells through exchange of Zn^2+^/H^+^ [3]. In contrast, ZIP transporters mediate the movement of zinc across the plasma membrane. Most of these ZIP transporters reside on the plasma membrane, facilitating influx of zinc from the extracellular matrix, though some members exist on the membranes of intracellular compartments, such as the Golgi apparatus (ZIP9 and ZIP13), intracellular storage vesicles (ZIP8, ZIP13, and ZIP14), and endoplasmic reticulum (ZIP7). Furthermore, intracellular zinc can also be associated with metallothionein or glutathione-bound protein within the cytosol. Storage of zinc in intracellular compartments is essential in order to facilitate rapid release of zinc, in the so-called “zinc-wave”, following influx of calcium and activation of mitogen-activated protein kinase (MAPK) [4]. This results in ZIP7-mediated zinc release from the endoplasmic reticulum [5] leading to cell-wide inhibition of tyrosine phosphatases and consequent prolonging of tyrosine kinase activity, several of which have been shown to be important in the development and progression of cancer.

ZIP7 plays a major role in regulation of zinc homeostasis, highlighted further by its ubiquitous expression throughout human cells [6]. ZIP7 regulates intracellular zinc release following its phosphorylation of residues S275 and S276 by protein kinase casein kinase 2 (CK2) [7]. This event results in a cascade of signalling events that leads to stimulation of zinc release from other storage compartments, as well as activation of multiple signalling pathways known to be important for growth in normal and disease states [8]. This zinc release will also cause activation of tyrosine kinases which have been directly implicated in breast cancer [9].

Clinically, it has been observed that *ZIP7* expression is frequently upregulated in breast cancer patients, particularly those who exhibit poor prognosis [10]. This trend was further observed in patients who have developed resistance to tamoxifen, as well as in our *in vitro* models of short-term and long-term tamoxifen resistance [8]. This overexpression is accompanied by significantly increased activated ZIP7 protein levels when compared to tamoxifen-sensitive models [11]. Taken together, these data infer that in tamoxifen-resistant cells there is an increased need for intracellular zinc, which subsequently drives more aggressive cell behaviors, especially those typically observed following development of resistance. The correlation of upregulated zinc release and aggression in these cases was further supported by significantly shorter relapse-free survival (RFS) in patients displaying increased *ZIP7* expression [11], as well as positive correlation between ZIP7 levels and spread to the lymph nodes [10].

Given the importance of zinc to cancer cell function, ZIP-transporter involvement in breast cancer has not only been limited to ZIP7-mediated release of intracellular zinc, but also extends to family members present on the plasma membrane. The expression and activity of one such transporter ZIP6 (SLC39A6), has long been associated with oestrogen receptor (ER)-positive breast cancer [12], being utilized in a clinical setting to identify luminal A breast cancer [13]. Similarly, the closely related family member ZIP10 has also been shown to be involved in breast cancer, with its expression shown to positively correlate with that of ER [10]. High levels of ZIP10 have also been linked to a more invasive phenotype and are commonly seen in breast cancer positive lymph nodes [14]. Importantly, to function as zinc channels, these transporters must become dimerized. Recently, our group demonstrated that these 2 family members have the ability to form a heteromer, essential to the cell migration and correct development of zebrafish embryos [15]. The same was true in breast cancer cells, with strong colocalisation of the ZIP6 and ZIP10 being observed in cells undergoing mitosis implying a role for ZIP6/ZIP10 heteromers in facilitating zinc influx to trigger mitosis [2]. This conclusion is concurrent with our group’s proposed model where extracellular influx of zinc through the ZIP6/ZIP10 heteromer is required in order for cells to enter mitosis. Activated ZIP6/10 heteromers can bind and convert signal transducer and activator of transcription 3 (STAT3) phosphorylated on tyrosine 705 (pSTAT3^Y705^) to pSTAT3^S727^ facilitating downstream activation of pStathmin^S38^ and subsequent microtubule reorganization required for cell division [2]. This also facilitates the release of ZIP6-associated Jarid1B, enabling its binding to and activation of pHistoneH3^S10^ to alleviate chromosome condensation for DNA replication [2]. Our group have previously shown that inhibition of this mechanism through ZIP6 or ZIP10 antibody binding can directly inhibit the mitosis of ER+ breast cancer cell lines *in vitro* [2].

Taken together these data imply that targeting ZIP6 and ZIP10, along with ZIP7, could provide an effective means of treating tamoxifen resistant (TAMR) breast cancer by impairing the availability of both intra- and extracellular zinc and attenuation of its subsequent downstream signalling. This should not only reduce mitosis but also have a significant effect on the aggressive phenotype often exhibited by these cells. In this study, we aimed to explore the functional relevance of ZIP7 and ZIP6/10 heteromers to TAMR breast cancer cells in comparison to non-resistant models of disease, in an attempt to validate anti-ZIP agents as a novel therapeutic target for resistant disease.

## Materials and methods

### Cell culture and materials

Anti-hormone responsive MCF-7 cells (AstraZeneca) were cultured as noted previously [8]. TAMR MCF-7 cells development has been previously described [16]. TAMR cells were maintained in phenol-red free RPMI 1640 (Life Technologies), supplemented with 5% charcoal stripped, steroid depleted foetal calf serum, 200 mmol/L *L*-glutamine, antibiotic cocktail (10 units/mL penicillin and 10 μg/mL streptomycin; Life Technologies), 2.5 μg/mL amphotericin B (Life Technologies) and 10^-7^ mol/L 4-OH-tamoxifen (Sigma Aldrich). For immunofluorescence and western blotting, cells were initially seeded at 1 × 10^5^ cells onto 0.17 mm glass coverslips or 60 mm dishes respectively, before being allowed to proliferate to 70–80% confluency prior to processing. In the case of immunofluorescence of nocodazole cell cycle synchronized cells, seeding and growth was as noted before cells were treated with 150 nmol/L nocodazole (Sigma Aldrich) in the presence or absence of anti ZIP6 antibody (in-house antibody) at increasing concentrations for 20 h and then processed. For proliferation cell counting assays, cells were seeded at 1 × 10^6^ cells/plate in 24-well plates and allowed to adhere for 24 h, at which point the first wells were counted using a Coulter Multisizer III (Beckman Coulter) and remaining cells treated with 4 μg/mL pZIP6 antibody (in-house antibody). To count, cells were trypsinised with 1 mL, 0.5% trypsin-EDTA (Gibco) and, once non-adherent, harvested into a syringe with a 25 G needle to ensure a single cell suspension. Resulting solution was then made up to 10 mL in isoton solution before counting was conducted. Selected wells were then counted at 24 h intervals until the completion of the experiment. All cells and experiments were incubated at 37°C and 5% CO_2_.

### Immunohistochemistry

Samples of MCF-7 and TAMR cells were fixed in 3.7% formaldehyde before incubation with pH 8 EDTA buffer in a pressure-cook microwave for 2 min at 950 W for optimal antigen retrieval. This was followed by blocking with serum-free blocking reagent (DAKO) and incubation with mouse monoclonal pZIP7 antibody (MABS1262, Merck Millipore) at 1:8,000 dilution for 1 h at room temperature in a humidity chamber. Slides were then briefly washed with phosphate buffered saline/Tween and incubated with secondary antibody [Mouse Envision labelled polymer-horse radish peroxidase (HRP), No. K4001, DAKO] for up to 1 h. 3’-3’-diamobenzidine (DAB) chromogen-substrate solution (DAKO) was used to visualize target protein and 0.05% (aqueous) methyl green was used to counterstain nuclei. Images were obtained using Olympus BH-2 microscope with multiple fields of view being imaged for analysis.

### Immunofluorescence

Cells were fixed using 3.7% formaldehyde (Sigma Aldrich) and processed as described previously [17]. Resulting coverslips were probed using primary antibodies (pZIP7 at 1:100, MABS1262, Merck Millipore; ZIP6 at 1:100, in-house antibody; pHistoneH3^S10^ 1:100, No. 9706S, Cell Signalling) for 1 h before incubation with Alexa Fluor secondary antibodies (goat anti-mouse Alexa Fluor 594, #A11032 Invitrogen; goat anti-mouse Alexa Fluor 488, #A10684, Invitrogen; goat anti-rabbit Alexa Fluor 594, #A11072, Invitrogen) at 1:1,000 dilution for 30 min. Coverslips were mounted on microscope slides using Vectorshield® mounting medium containing DAPI (Vector Laboratories), prior to sealing with clear nail varnish. Slides were subsequently imaged using a 63× oil immersion lens on a Leica RPE automatic microscope. Image acquisition was through use of Openlab software for Macintosh overall survival (OS) and resulting images processed using FIJI software.

### Flow cytometry

Free cytoplasmic zinc was measured using FluoZin-3AM (Invitrogen), a cell permeant, zinc-fluorescent dye. Cells at 70–80% confluency in 35 mm dishes were trypsinised, prior to 30 min incubation at 37°C with 5 μmol/L FluoZin-3AM. Following this, cells were placed in fresh, FluoZin-3AM free media for 30 min before assessment by flow cytometry. In order to quantify results, basal levels of fluorescence were evaluated using method previously outlined [18]. Briefly, live cells were treated with zinc and 50 μmol/L of the chelating agent TPEN in order to establish baseline fluorescence (*F*), as well as minimum fluorescent intensity following chelation (*F*_0_) and maximum fluorescent intensity after treatment with 100 μmol/L zinc plus 10 μmol/L sodium pyrithione (*F*_max_). The following formula was then used to determine zinc concentration (where *K*_D_ is 15 nmol/L [18]):

$$[Zn]=K_{D}\left(\frac{F-F_{0}}{F_{\text{max}}-F_{0}}\right)$$

Analysis was conducted on live cells using BD FACSVerse™ flow cytometer and resulting data analysis using FIJI software.

### Western blotting

Western blotting samples were prepared as previously described [9]. Following SDS-PAGE, the following primary antibodies were used: total ZIP7 (19429-1-AP, Proteintech™); pZIP7 (MABS1262, Merck Millipore); pAKT^S473^ (No. 9271, Cell Signalling). All were used at 1:1,000 dilution. HRP-conjugated β-actin (A3854, Sigma Aldrich) used at 1:10,000 was utilized as loading control. Densitometry analysis was performed using Alpha DigiDoc software, with results being normalized to their respective loading control.

### Kaplan-Meier plotter and GEPIA analysis

Exploration of matched tumour *versus* normal breast tissue levels of ZIP family members was conducted using publicly available online gene expression profiling interactive analysis (GEPIA2, http://gepia2.cancer-pku.cn/#index; [19]). All results show matched gene expression data derived from The Cancer Genome Atlas (TCGA) and the Genotype-Tissue Expression (GTEx) databases, with data filtered to show only differential gene expression relationships. Correlation of *ZIP7* and *ZIP6* expression data and survival in breast cancer patients was assessed using publicly available online KmPlot database (www.kmplot.com) [20]. Data were generated by using Affymetrix microarray expression of messenger RNA (mRNA), utilizing JetSet ptimized probes for ZIP7 (202667_s_at) and ZIP6 (202088_at) in tumour samples from breast cancer patients. Patient cohorts were defined by “auto-selected” cutoffs and examined for overall and RFS. Further analysis restricted patient groups to those treated with systemic endocrine therapy and those treated with tamoxifen only. Both analyses were conducted including patients who had received chemotherapy.

### Statistics

Statistical analysis of data was conducted using an independent t-test or ANOVA with Dunnett *post-hoc* tests. All values are expressed as the mean of a minimum three experiments, across biological replicates (n ≥ 3) with error bars representing standard error of the mean (SEM). Results were deemed significant when *P* < 0.05.

## Results

### ZIP7 is upregulated in breast cancer cells following development of tamoxifen resistance

**Figure 1:**
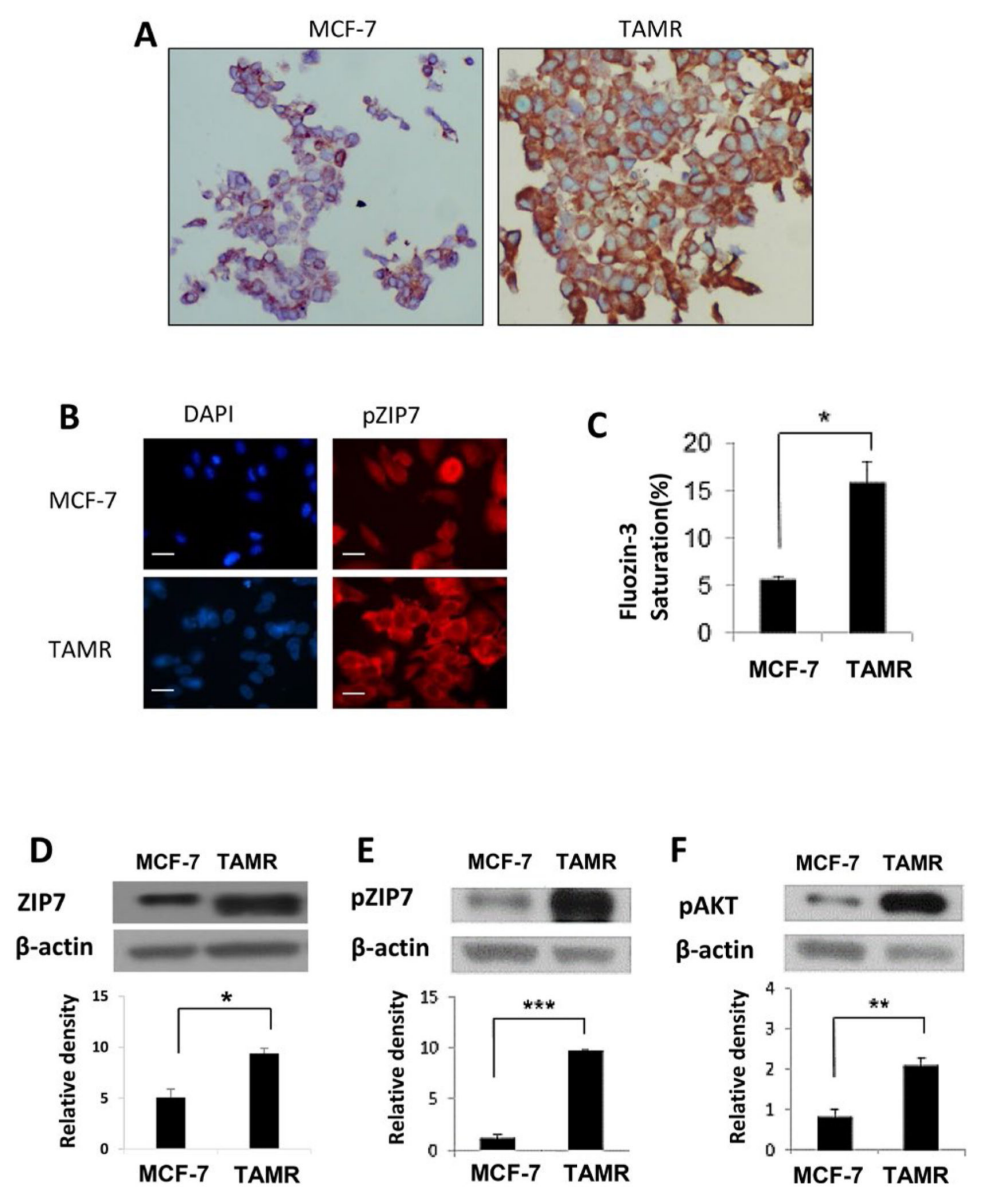
Comparison of ZIP7 levels and activity between anti-hormone responsive MCF-7 and resistant TAMR cell lines. (A) Representative images from immunohistochemistry comparing total ZIP7 levels (brown) between MCF-7 and TAMR cells. Cell nuclei were counterstained with methyl green (blue). Images were obtained at 40× magnification. (B) Immunofluorescence MCF-7 and TAMR cells probed for pZIP7 (red), alongside DAPI (blue). Pictures are representative of individual assessments from replicate experiments (*n* = 3). Images were obtained using Leica Microscope with a 63× oil immersion lens. Scale bars represent 10 μm. (C) Comparative flow cytometry analysis for levels of free cytoplasmic zinc, as represented by Fluozin-3 fluorescent intensity, between MCF-7 and TAMR cells (*n* = 3). Examples of western blots of total ZIP7 (D), pZIP7 (E), and major downstream target pAKTS473 (F) including respective densitometry to evaluate protein level signalling changes between MCF-7 and TAMR cells (*n* = 3). For all data, graphs show mean values plus error bars representing SEM; *P < 0.05, **P < 0.01, ***P < 0.001

We first wished to examine the relationship between activated ZIP7 and the development of resistance to tamoxifen. Utilizing an MCF-7 derived *in vitro* model of acquired anti-hormone resistance that mimics clinical progression to this disease state (TAMR cells [16]), as well as the tamoxifen-sensitive MCF-7 cells, basal levels of ZIP7 were compared using immunohistochemistry. Reflecting previous clinical data, both cell lines displayed expression of the ZIP7 protein, with expression being significantly amplified in the TAMR cells when compared to the anti-hormone sensitive MCF-7 cells (Figure 1A). Given the importance of activated ZIP7 to its function, we next used immunofluorescence to examine the level of activated ZIP7 using our specific ZIP7 monoclonal antibody that only recognizes residues S275 and S276 of ZIP7 when they are activated by phosphorylation [9]. How active protein levels vary between the 2 models was also examined. As with immunohistochemical analysis, phosphorylated ZIP7 levels show marked increase in TAMR *versus* MCF-7 cells (Figure 1B). Moreover, pZIP7 levels were consistent with this observation, with anti-hormone resistant cells displaying significantly increased activity. Indeed, examination of positive cells showed that almost all TAMR cells showed high positive staining for pZIP7, whilst hormone-responsive MCF-7 cells showed significantly lower levels suggesting that TAMR cells are all actively utilizing ZIP7-mediated signalling for their growth.

Taken together these results infer that development of anti-hormone resistance results in an increase in ZIP7 mediated cell signalling due to augmented intracellular zinc release from the endoplasmic reticulum. Consequently, flow cytometry was used to assess the levels of zinc within our chosen cell models using FluoZin-3AM; a sensitive, Zn^2+^ specific fluorescent dye. As with pZIP7 activity, TAMR cells showed a significant increase (*P* < 0.05) in saturation of FluoZin-3AM compared to MCF-7 cells, reflecting approximately 3-times as much free zinc within anti-hormone resistant cells (Figure 1C). To further examine the functional relevance of this increased zinc release, we next used western blotting to examine pAKT^S473^ activity, as well as protein level changes in ZIP7 expression/activity. Previous studies have demonstrated AKT as a major downstream target of ZIP7-mediated Zn^2^+ release from the endoplasmic reticulum in breast cancer, with its activity resulting in sustained cell survival and anabolic cell growth [9]. As expected, total ZIP7 and pZIP7 are both significantly increased (*P* < 0.001) in TAMR cells, with a 2- and 10-fold change noted respectively (Figure 1D and E). This result infers that, not only do anti-hormone resistant cells upregulate ZIP7, but these cells display a state of ZIP7 hyperactivity which would justify the observed increases in cytosolic zinc in these cells. Preferential activation of pZIP7 in TAMR cells was concomitant with significantly increased (*P* < 0.01) pAKT^S473^ activity *versus* anti-hormone sensitive cells (Figure 1F). This observation is consistent with our previous work showing that ZIP7 knockdown and zinc chelation leads to a significant reductions in pAKT^S473^ activity [8]. These data demonstrate a functional consequence for increased ZIP7 levels and activity in anti-hormone resistant breast cancer *versus* sensitive cells. Tamoxifen resistance not only leads to increased levels of ZIP7 but seemingly its hyperactivation, resulting in increased release of intracellular zinc from the endoplasmic reticulum and thus modulated signalling of known zinc regulated kinases. Coupled with the variation in ZIP7 expression between anti-hormone sensitive and resistance cell lines, this further infers that ZIP7 signalling becomes constitutively active following development of tamoxifen resistance, potentially becoming a key driver of proliferative signalling in these cells.

### Assessing the *in vitro* role of ZIP6 and ZIP10 in anti-hormone resistant breast cancer

**Figure 2:**
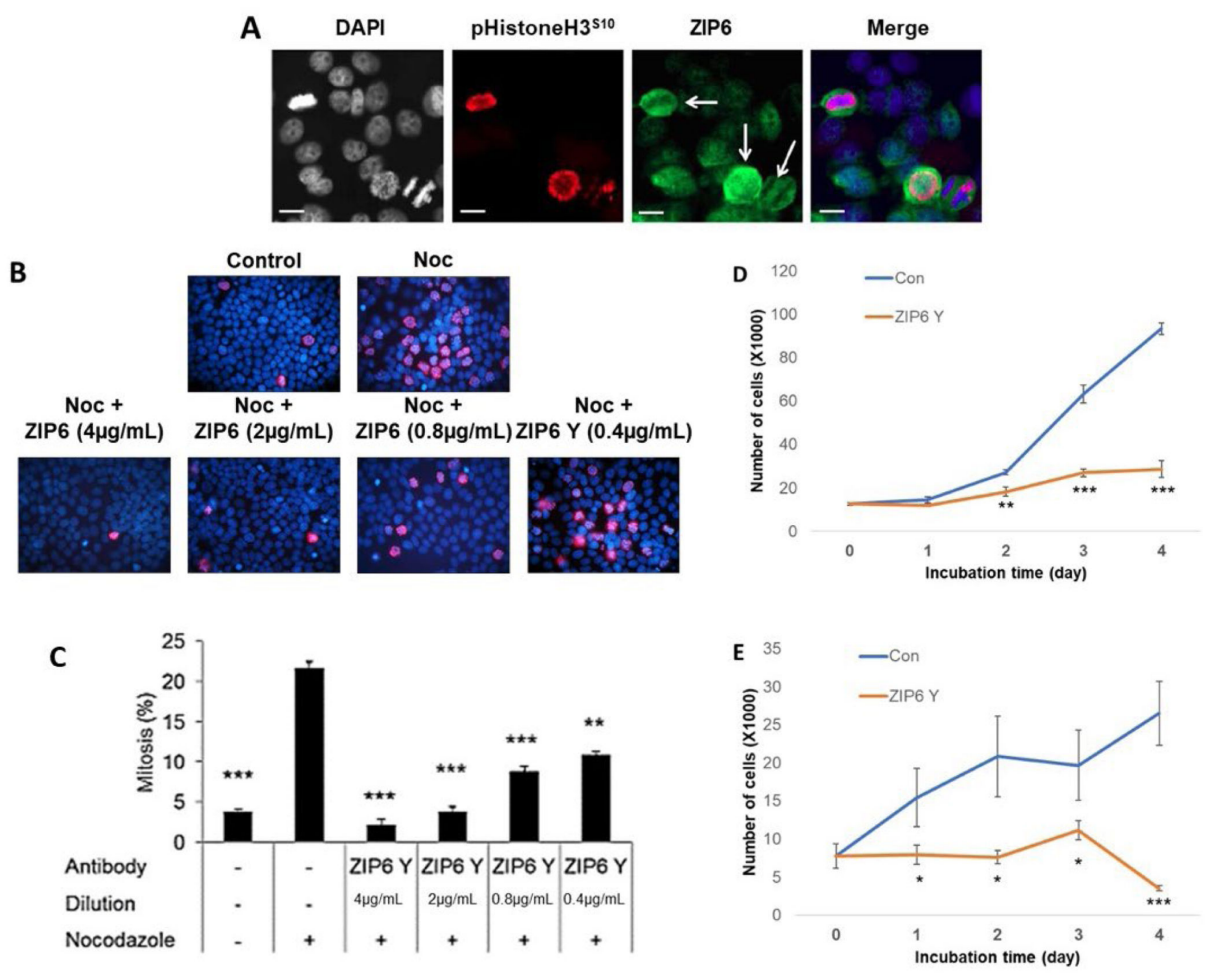
Assessment of ZIP6 activity in regulating mitosis of cancer cells *in vitro*. (A) Immunofluorescent evaluation of phosphorylated ZIP6 (green) activity in MCF-7 cells, in comparison with pHistoneH3^S10^ (red) as a marker for cells actively undergoing mitosis, plus additional co-stain for cell nuclei with DAPI (blue). White arrows identify cells undergoing active mitosis. Images are representative of multiple assessment (*n* = 3). Scale bars represent 10 μm. (B) Cells were treated with 100 nmol/L nocodazole, in the presence or absence of anti-ZIP6 antibody across a dose range, for 20 h prior to examination of mitotic changes through counting cells positive for immunofluorescent detection of pHistoneH3^S10^ (red). Cells nuclei were co-stained with DAPI (blue). Images are representative of replicate data (*n* = 3). Quantification of results is shown in (C). Proliferation of MCF-7 (D) and TAMR (E) cells were assessed ± treatment with 4 μg/mL ZIP6 antibody. Cells were counted at 24 h intervals following initial treatment and at day 0 (*n* = 3). All graphs display mean values with error bars representative of SEM; *P < 0.05, **P < 0.01, ***P < 0.001. Noc: nocodazole; Con: Control

Having demonstrated the importance of intracellular zinc release to TAMR cells, we next wanted to evaluate the functional relevance of extracellular zinc influx, particularly that mediated by ZIP6. Our group have previously demonstrated that ZIP6/ZIP10 heterodimers are essential to trigger the process of mitosis through regulating influx of Zn^2+^ and its subsequent modulation of pSTAT3 signalling [2]. We wished to expand on this work by exploring the functional relevance of this in tamoxifen responsive cells in comparison to those that had developed resistance. As such, we began by exploring the presentation and distribution of ZIP6 in cells using immunofluorescence (Figure 2A). Generally, cells display a low level of *ZIP6* expression, with most being primarily localized to the endoplasmic reticulum. However, approximately 5% of cells showed ZIP6 presentation on the plasma membrane which was confined to cells undergoing mitosis, as confirmed by DAPI staining and positive co-staining with pHistoneH3^S10^. These cells also displayed the characteristic rounding associated with early mitotic events; a process known to be dependent on ZIP6-mediated Zn^2+^ influx. Taken together, these data reflect the need of this transporter to relocate to the cell periphery in order to trigger mitosis.

To further examine the relevance of ZIP6 to breast cancer mitosis, we explored whether endogenous division could be inhibited by the use of an anti-ZIP6 targeting antibody. We decided to utilize nocodazole to increase the number of cells in mitosis, in combination with increasing doses of ZIP6 antibody, to evaluate its role in driving mitotic events. Nocodazole blocks microtubule polymerization, thus inhibiting progression through mitosis. Following 20 h in the presence of nocodazole, the number of pHistoneH3^S10^ positive cells increased significantly *versus* non-blocked control cells (Figure 2B and C), representative of the increased percentage of cells in mitosis. The presence of the antibody significantly attenuated the number of mitotic cells at all concentrations, with decreasing concentration lessening the inhibition in a dose dependent manner. These data suggest that correct ZIP6 function is essential in allowing cells to progress through mitosis.

The low percentage of mitotic cells in the absence of nocodazole could imply endogenous mitosis is limited in these cells. As such, despite significantly affecting DNA-condensation, as reflected by reduced pHistoneH3^S10^, the functional effects of this ZIP6 inhibition may be very limited. We thus wished to examine how these protein level changes affected overall cell proliferation through performing cell counting analysis following treatment with our ZIP6 antibody. Cells were allowed to grow for 24 h following seeding, prior addition of the antibody to ensure any changes in cell number were as a result of impaired division and not potential disruptions in adhesion. Cells were subsequently counted at 24 h intervals including at the point of antibody addition (0-day timepoint). MCF-7 cells showed a significant reduction (*P* < 0.01) in proliferation following 2-day growth in the presence of the antibody (Figure 2D). This inhibition was sustained for the remainder of the time-course, with greater effects noted later in the assay. Interestingly, significant inhibition seemed to coincide with initiation of log-phase growth in the untreated cells. Given the clinical report that anti-hormone resistant cells exhibit increased aggression and growth rates compared to sensitive cells [21], we hypothesized that our TAMR model would show increased effects of inhibition following ZIP6 inhibition. Therefore, we repeated the proliferation experiment using TAMR cells (Figure 2E). As with tamoxifen sensitive MCF-7 cells, ZIP6 inhibition was sufficient to induce a significant reduction in proliferation throughout the duration of the assay. However, suppression of proliferation was noted at an earlier timepoint (1-day *versus* 2-day) in the TAMR cells compared with MCF-7 cells, likely due to exponential proliferation and thus increased mitosis at this point in the former. Generally, the inhibitory effects on TAMR cells were greater, inferring a preferential efficacy of anti-ZIP6 treatment in anti-hormone resistant cells. More work is needed to confirm this hypothesis, however preliminary studies *in vivo* have suggested preferential inhibition of tamoxifen-resistant tumours over anti-hormone sensitive disease.

### Evaluation of ZIP transporters in clinical samples through use of online databases

**Figure 3:**
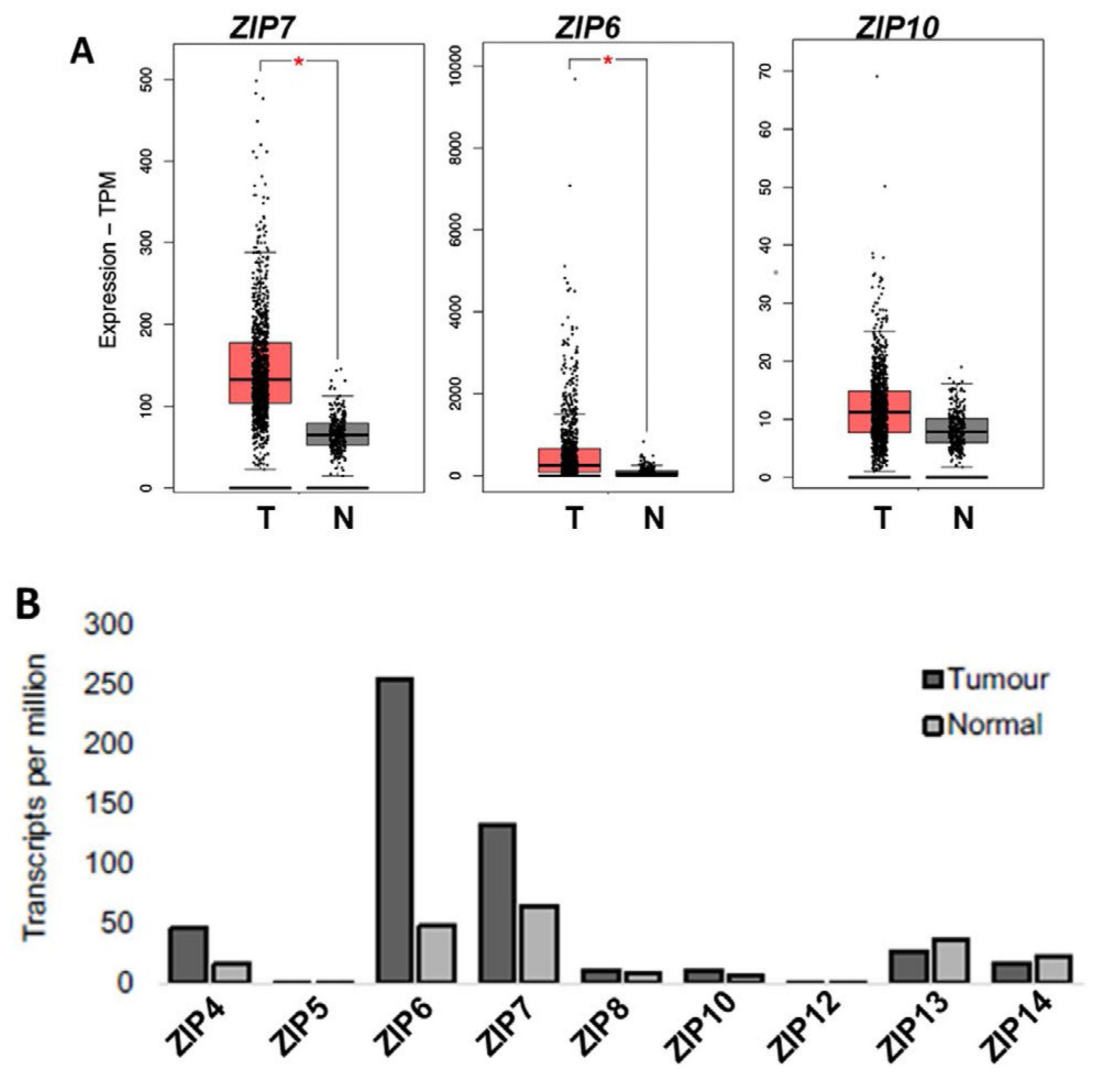
Differential expression profiles of ZIP family members in paired cancerous *versus* normal breast tissue, including focused analysis of ZIP4, ZIP7, ZIP6, and ZIP10. (A) Expanded analysis of LIV-1 subfamily members of ZIP-family transporters in matched normal *versus* tumour patient samples. (B) Focussed analysis of matched tumour and normal breast tissue levels of ZIP4, ZIP7, ZIP6, and ZIP10. Red and grey boxes represent cancerous (T) and matched normal (N) tissue, respectively. All plots were produced by GEPIA 2 server [19] and describe matched gene expression data derived from TCGA and GTEx databases. Data was filtered to show only differential gene expression relationships with a *P*-value threshold of 0.01. Log2FC cut-off was designated as 1. Patient numbers (*n*) in cancerous (T) and normal (N) cohorts were 1,085 and 291 respectively. TPM: transcripts per million

Having demonstrated the potential of ZIP-targeting therapeutics *in vitro*, we wished to explore the relevance of these agents in a clinical setting through the use of online digital collections of patient data. The aim was to enable identification of potential subgroups of breast cancer patients that would most benefit from clinical inhibition of zinc signalling, beyond those who may have developed tamoxifen resistance. Using the GEPIA 2 server [19], we began by undertaking an expanded analysis of all 9 human members of the LIV-subfamily of ZIP transporters (Figure 3A). Across the family members only ZIP4, ZIP6 and ZIP7 showed any significant variation between normal and tumour tissue, with all showing a significant increase in tumours. ZIP4 is primarily responsible for the intake of zinc in the gut to maintain body zinc homeostasis by shuttling zinc across the plasma membrane to be muffled and stored in the endoplasmic reticulum, where its subsequently released by ZIP7 [22]. This change implies increased use of zinc stores which results in zinc deficiency in tumour cells and thus upregulation of ZIP4 to replenish intracellular zinc stores. This further highlights the importance of upregulated zinc homeostasis in cancer cells, while inferring a potential upstream mechanism for sustained ZIP7 activity and intracellular zinc release. Interestingly, the levels of ZIP6 and ZIP7 were comparable in normal tissue but ZIP6 expression in tumours was significantly higher than that of ZIP7.

Given this variation between family members, we performed a focused analysis on how ZIP4, ZIP7 and ZIP6 expression compared between matched sets of tumour and normal breast tissue taken from patients (Figure 3B). Given the importance of the ZIP6/ZIP10 heteromer, we expanded our evaluation to include ZIP10 in these samples as a means of assessing its future potential for therapeutic development. When compared to normal breast tissue, ZIP4, ZIP7, and ZIP6 showed a significant increase (*P* < 0.01) in expression in tumour cells. Contrastingly, ZIP10 showed no significant difference in expression between normal and tumour samples, possibly suggesting it as a lesser partner of the heteromer, with ZIP6 homo- and heterodimers being of greater importance to extracellular influx of zinc.

**Figure 4:**
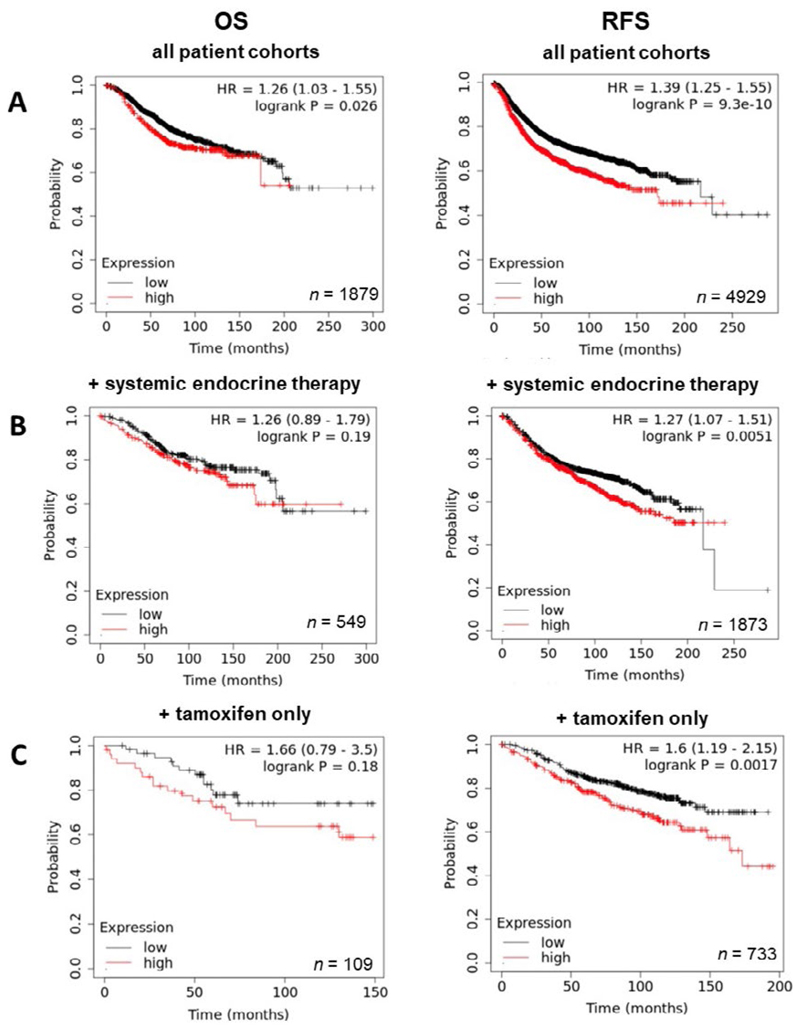
Kaplan-Meier plots describing *ZIP7* mRNA expression relationship with OS and RFS, in breast cancer patients. All plots were generated using KMPlot [20] with expression cohorts defined by “auto-selected” cut-offs. Probe sets were optimized via “JetSet” function. Patient cohorts were progressively focused from unrestricted analysis (A), initially into those receiving systemic endocrine therapy (B), before isolating those specifically treated with tamoxifen (C). Hazard ratios (HRs) and significance values (*P*) are indicated on graphs, with the number of patients included in each analysis (*n*) noted

Having demonstrated the upregulation of ZIP7 and ZIP6 in matched tumour samples, we next wanted to examine how this effect translates to prognosis and disease progression. Therefore, Kaplan-Meier plots were generated to correlate expression with OS and RFS. Generally, high expression of ZIP7 in patients significantly correlated with poorer prognosis, with patients exhibiting lower rates of OS and RFS (Figure 4A). Interestingly, OS in patients following systemic endocrine therapy was unaffected by levels of ZIP7, however RFS was significantly lower in these patients (Figure 4B). When this subcategory was limited to tamoxifen only treatment, significantly reduced RFS was noted, with OS unaffected (Figure 4C). These data imply that ZIP7 is not only worse for patient prognosis but may be indicative of poor response to tamoxifen and may even suggest a potential mechanism for development of resistance.

**Figure 5:**
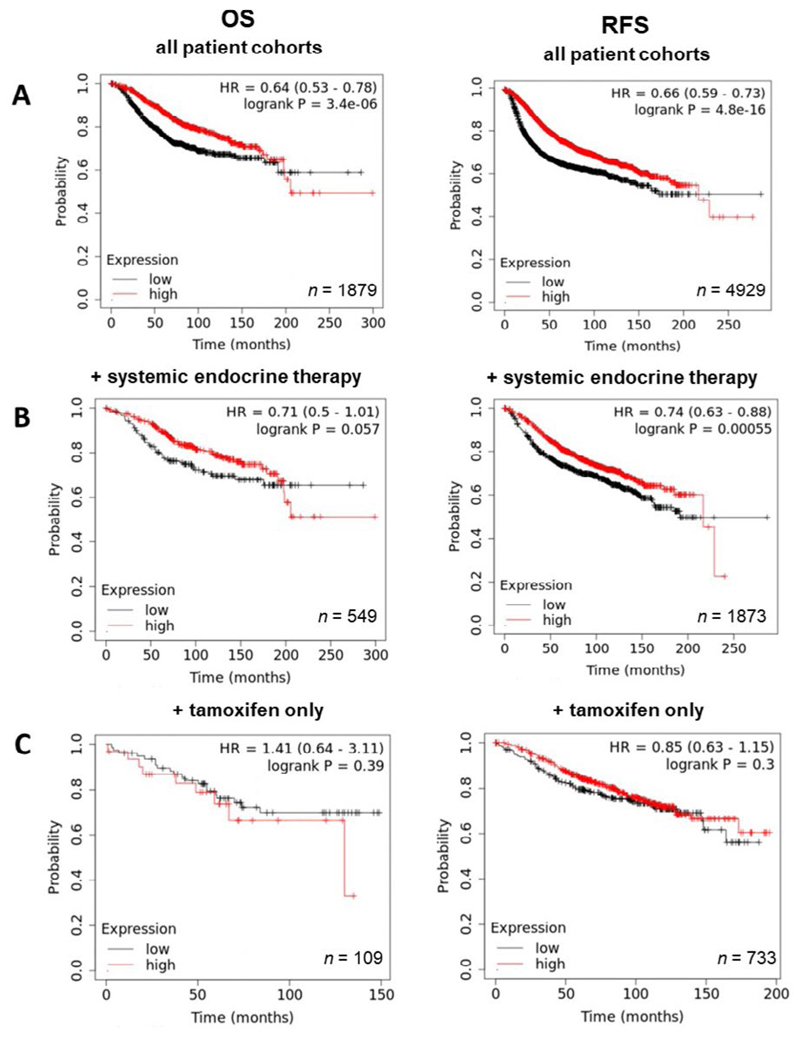
Kaplan-Meier plots describing *ZIP6* mRNA expression relationship with OS and RFS, in breast cancer patients. All plots were generated using KmPlot [20] with expression cohorts defined by “auto-selected” cut-offs. Probe sets were optimized via “JetSet” function. Patient cohorts were progressively focused from unrestricted analysis (A), initially into those receiving systemic endocrine therapy (B), before isolating those specifically treated with tamoxifen (C). Hazard ratios (HRs) and significance values (*P*) are indicated on graphs, with the number of patients included in each analysis (*n*) noted

By contrast, exploration of ZIP6 revealed a significantly positive correlation between expression and prognosis, with levels of overall and RFS being higher in patients with high ZIP6 levels (Figure 5A). Further analysis based on treatment showed that, while in tamoxifen only (Figure 5C) patients ZIP6 expression had no significant effect on overall or RFS, high ZIP6 levels significantly improved RFS in patients given systemic endocrine therapy (Figure 5B), though OS was unaffected. Effects of ZIP10 expression were limited with only a slightly significant negative correlation being observed with OS (data not shown). These results seem to suggest that presentation of ZIP6 is better for general patient prognosis and does not impact on standard methods of care. However, these results do not specifically focus on patients that have developed anti-hormone resistance. Given the data presented here, we hypothesize that this subset of patients would typically exhibit higher levels and activity of ZIP6 and would therefore benefit from anti-ZIP6 therapeutics.

**Figure 6:**
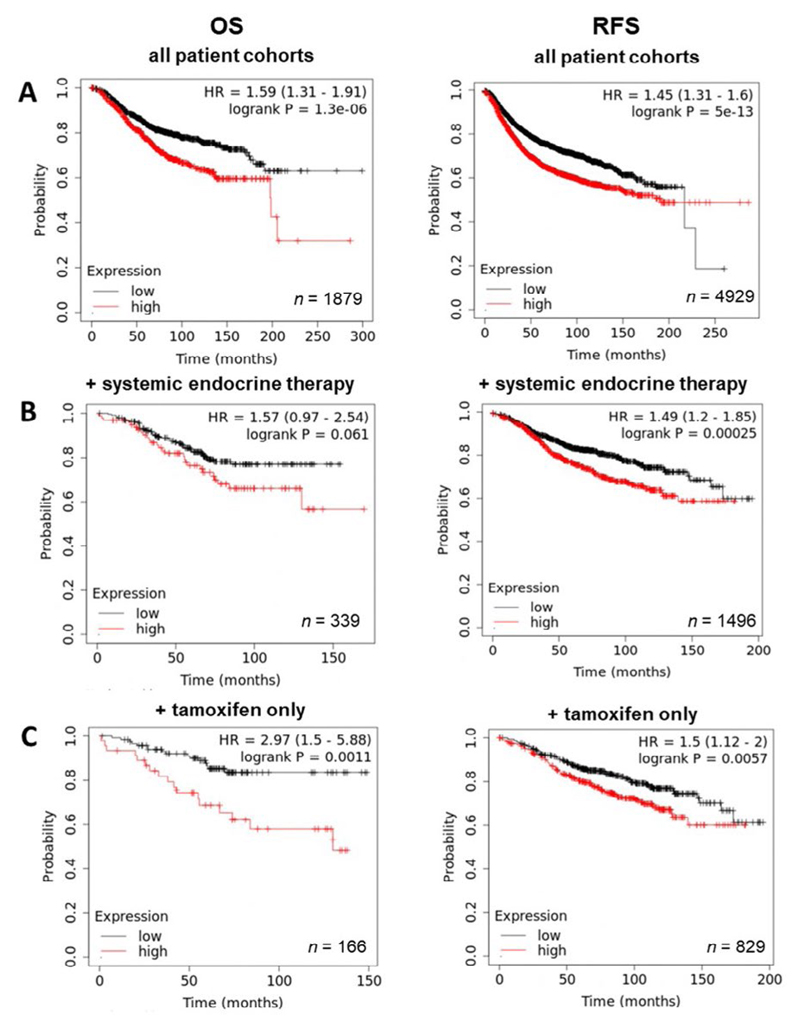
Kaplan-Meier plots describing ZIP4 mRNA expression relationship with OS and RFS, in breast cancer patients. All plots were generated using KmPlot [20] with expression cohorts defined by “auto-selected” cut-offs. Probe sets were optimized via “JetSet” function. Patient cohorts were progressively focused, initially to “include” those non-specific systemic endocrine therapy (B), before isolating those specifically treated with “tamoxifen only” (C). Hazard ratios (HRs) and significance values (*P*) are indicated on graphs, with the number of patients included in each analysis (*n*) noted

Given the significant increase in ZIP4 expression in tumour samples noted (Figure 3), the relevance of this to patients was explored using KmPlot. Generally, patients with high levels of ZIP4 had a significantly worse prognosis, with both OS and RFS being reduced (Figure 6A). RFS was also significantly reduced in the high-expressing cohort who received systemic endocrine therapy, while no significant difference in OS was observed (Figure 6B). However, when this analysis was confined to patients who only received tamoxifen, high ZIP4 significantly correlated with poorer OS, as well as RFS (Figure 6C). Together, these data imply that ZIP4 plays a significant role in breast cancer progression. Moreover, the result observed with tamoxifen treated patients could imply ZIP4 as a major marker for poor response to tamoxifen treatment, potentially contributing to the development of resistance. As noted above, ZIP4 increase may also be an indicator of zinc deficiency and so further experimental evaluation is needed to illuminate the role of ZIP4 in this context.

## Discussion

In recent years, the relevance of zinc homeostasis in relation to cancer has been illuminated, though its therapeutic potential largely remains unexplored. However, fundamental research into the mechanisms of zinc signalling is beginning to highlight just how beneficial targeting zinc transporters may prove as anti-cancer agents for a host of different cancer types.

**Figure 7:**
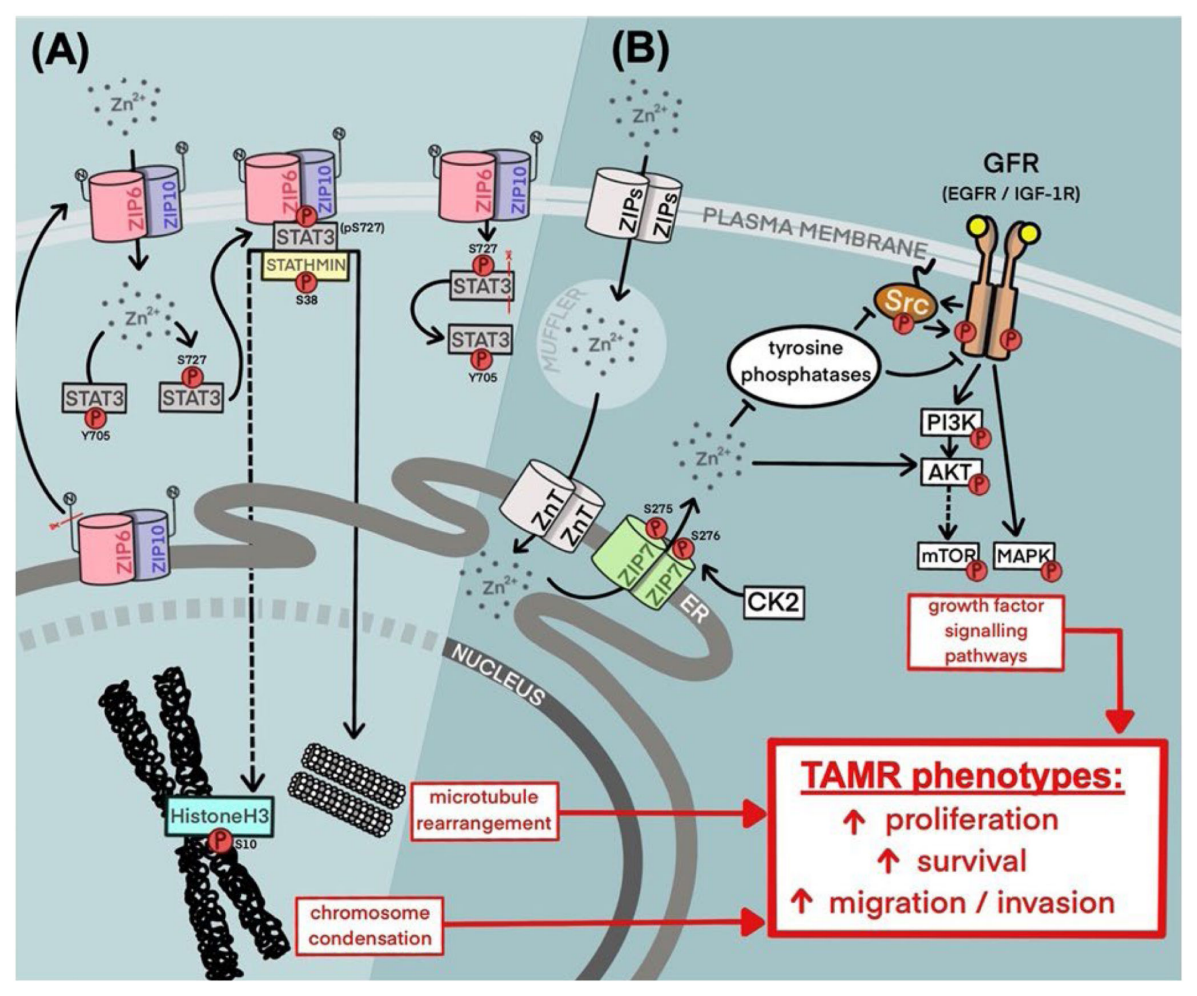
Summary of current knowledge of ZIP6, ZIP10, and ZIP7 contribution to tamoxifen-resistant phenotypes in breast cancer. (A) A mechanism for ZIP6:ZIP10 heterodimer-mediated induction of mitosis. Red lines represent the sites of protein cleavage. (B) A mechanism for stimulation of growth factor signalling via ZIP7-mediated intracellular zinc transport. cdh1: E-cadherin; elp1: elongator acetyltransferase complex subunit 1; ER: endoplasmic reticulum; CDK2: cyclin-dependent kinase 2; PI3K: phosphoinositide-3-kinase; mTOR: mammalian target of rapamycin; Src: proto-oncogene tyrosine-protein kinase Src

The study presented here has focused on the relevance of ZIP7 and ZIP6 as potential therapeutic targets within breast cancer, with a specific focus on TAMR; a growing obstacle to patient outcome [23]. Herein, we have demonstrated a preferential upregulation of ZIP7 within a model of tamoxifen resistance when compared to a responsive model. This was accompanied by hyper-phosphorylation of this transporter resulting in consequential release of zinc from intracellular stores, as well as increased activation of key downstream signalling partners. Interestingly, the pathways regulated in this way (summarized in Figure 7) are typically those associated with increased cell survival and movement to a more aggressive phenotype, as previously demonstrated in clinical data [9]. This correlates with the behaviors exhibited by these cells, showing both increased growth rates and migration *versus* the responsive cell model. Indeed, this also translates to patient data where patients who develop tamoxifen resistance are more likely to present with lymph-node involvement and shorter RFS periods. Additionally, unlike our tamoxifen sensitive model, TAMR cells are almost all positive for active ZIP7. Taken together, these observations may highlight ZIP7 as a key driver of resistant cell signalling, with its inhibition providing a means by which to successfully attenuate disease recurrence and potentially reduce metastatic disease.

The prevalence of activated ZIP7 in TAMR cells may also provide a rationale for its use as a predictive marker for the development of therapeutic resistance. We hypothesize that this change in ZIP7 drives resistance in these cells through modulation of survival pathways, namely AKT. Activity of AKT has long been associated with cell survival, with its function being directly linked to multi-drug resistance across a number of cancers, including breast [24]. In addition, previous work by our group has demonstrated that increased intracellular zinc release in breast cancer cells by active ZIP7, can also lead to decreased glycogen synthase kinase-3β (GSK3β) through inhibitory phosphorylation, further amplifying AKT driven cell survival [9].

ZIP7 may also drive this *de novo* tamoxifen resistance through modulation of growth factor receptor pathways linked to resistance. It has been demonstrated that ligand-independent activation of ER can be achieved through enhanced MAPK signalling, enabling desensitization to anti-hormone agents [25]. This enhancement also results in increased signalling downstream and independent of ER activation, essentially bypassing the need for upregulated oestrogen signalling. This method of resistance has been shown to be further amplified by activation of upstream growth factor receptors, namely insulin-like growth factor receptor (IGFR) and epidermal growth factor receptor (EGFR) [26, 27]. The increased activity of both receptors independent of ER-signalling provide an indirect means of cell proliferation and survival even in the presence of anti-endocrine agents, with their activity being previously noted in our TAMR models, despite the developed resistance [16]. Our previous work has shown that increased cytoplasmic zinc as a result of activated ZIP7 causes significant augmentation of MAPK signalling, as well as direct activation of IGFR and EGFR [9]. Consequently, the hyperactivation of ZIP7 and increased release stored zinc in TAMR cells, as presented here, could represent a major mechanism by which *de novo* anti-hormone resistance can be achieved.

Exploration of patient data revealed significant increases in both ZIP7 and ZIP6, as well as the family member ZIP4, in tumour samples compared to matched normal breast tissue. This supports the relevance of ZIP-transporter activity as a significant factor in breast cancer. Interestingly, ZIP6 levels in cancer are significantly higher than levels of ZIP7. Given the aberrant growth of cancer cells, this observation is consistent with our model of ZIP6 driving tumour cell mitosis, as all cells would require ZIP6-mediated influx of zinc for division, whereas ZIP7 activity correlates with more invasive behaviors thus limiting its overexpression to a subset of more aggressive cells (Figure 7). However, levels of ZIP10 are significantly lower than that of ZIP6. Having previously demonstrated a mechanism by which ZIP6/ZIP10 regulates zinc influx to initiate mitosis [2], these data infer that ZIP10 may play a secondary role and that this mechanism may be primarily driven by ZIP6. Indeed, our group have previously shown that ZIP10 levels significantly increase following knockout of ZIP6 [2], suggesting a mechanism of compensation which further infer a supplementary role of ZIP10 in ZIP6-regulated zinc signalling.

Further examination of the ZIP transporters revealed significant trends in expression *versus* patient outcome. Generally, high ZIP7 levels correlated with a poorer patient outcome, as well as significantly affecting RFS in patients treated only with tamoxifen. These data mirror the imbued cell traits exhibited by *ZIP7* over-expressing cells previously noted; namely increased cell survival, EMT and metastasis [9]. Moreover, worse RFS in the patient group treated only with tamoxifen seems to further solidify our hypothesis that ZIP7 could be predictive of development of resistance and could explain why we see the trends observed.

Similarly, high ZIP4 expression correlated with a poorer prognosis for patients. Interestingly, previous work has demonstrated that dysregulation of zinc homeostasis as a result of increased ZIP4 activity, can promote the formation of brain metastasis from triple-negative breast cancer cells [28]. This study also found that this increased cellular zinc can increase tumourgenic capacity of breast cancer stem cells. If this observation holds across breast cancer subtypes, this could explain the poorer prognosis of the high ZIP4 cohort, with this tumour type displaying increased aggressive tendencies and metastatic potential. The noted effect on stem cells may also provide a rationale for the observed effects in tamoxifen treated patients, owing to the inherent drug resistance of this subpopulation.

In contrast, high levels of ZIP6 seem indicative of improved prognosis, with significant increases in overall and RFS levels in these patients. Given the role we have shown for ZIP6-mediated zinc influx in driving mitosis [2], we hypothesize that the noted improvements could be as a result of increased responsiveness to broad scale chemotherapy owing to increased growth rates of ZIP6-high tumours. Indeed, a focused analysis of KmPlot data examining solely chemotherapy treated patients shows a very significant positive relationship between high ZIP6 levels and response to treatment. This could imply the utilization of ZIP6 as a biomarker for chemo-responsiveness until the development of alternative therapeutic pathways that take us away from traditional chemotherapy. Interestingly, this could also provide a prognostic marker for those breast tumours which lack effective targeted therapies, for example in triple-negative disease. Unfortunately, this sort of analysis is not possible on patients exhibiting resistant disease, wherein we would expect levels of ZIP6 to be amplified and driving mitosis. Although this could make such cells increasingly susceptible to traditional chemotherapy, we believe that this expression drives enhanced proliferation and aggression in these cells thus its inhibition is essential to improving long-term RFS. The upregulation of ZIP6 also provides a unique and effective molecular target in TAMR cells, as demonstrated by the increased effectiveness of ZIP6 inhibition in TAMR *versus* anti-hormone sensitive cells presented here. The variation in trends observed between ZIP7 and ZIP6 further serve to highlight the importance of patient stratification for any future developments of anti-ZIP agents.

Although the specific transporters highlighted display the potential to be effective therapeutic targets, this family of transporters do not exist in isolation, rather they act in tandem with the ZnT family transporters to modulate zinc homeostasis in cancer cells, the overexpression of ZnT2 in particular [3, 29]. This transporter buffers cytoplasmic zinc by actively moving it into vesicles thus protecting cells from zinc-induced cytotoxicity [30]. Similarly, breast cancer cells have been shown to upregulate metallothionein which can bind free zinc thus reducing toxicity by sequestering its availability for cell signalling [31]. Consequently, these proteins may play a key role in sustaining cell viability, and thus signalling, in the presence of upregulated cytoplasmic zinc as a direct result of increased ZIP family transporter activity. This network of regulation also includes several members than can modulate zinc signalling in a more indirect manner. For example, the zinc sensing receptor GPR39 causes calcium influx in response to extracellular zinc [32]. Indeed, this receptor has been implicated in driving cancer cell growth [33] and has been shown to be upregulated in TAMR cells in contrast to responsive cells [34]. From these studies, we would hypothesise that GPR39 may contribute to ZIP7-mediated tamoxifen resistance by increasing calcium influx leading to enhanced intracellular zinc release. Although no data is currently available, evaluation of the relationship between ZIP6/ZIP10 and GPR39 during mitosis could provide new insights of clinical relevance. Taken together with the data included here, these studies further highlight the importance of the zinc homeostatic network and may provide a basis from which to enhance the efficacy of novel anti-ZIP family transporter agents and expansion to further enhance patient prognosis or overcome therapeutic resistance.

In summary, we have explored the relevance of both ZIP7 and ZIP6 as therapeutic targets in anti-hormone resistant breast cancers, with both our *in vitro* studies and examination of clinical data highlighting the potential of their targeting in the context of tamoxifen resistance. This has shown the wider application of anti-ZIP7 agents as a breast cancer therapeutic, with broad scale applicability prior to and following development of resistance. Furthermore, our results suggest that anti-ZIP6 agents could be a valid approach for intervention in endocrine resistant patients, with increased preference to this tumour type over those that are anti-hormone responsive. However, work is needed to identify patient groups that would most benefit from this treatment and impair the benefits ZIP6 may have upon response to other therapeutic options.

## Abbreviations

EGFR: epidermal growth factor receptor
ER: oestrogen receptor
GEPIA: Gene Expression Profiling Interactive Analysis
IGFR: insulin-like growth factor receptor
KmPlot: Kaplan-Meier Plotter
MAPK: mitogen-activated protein kinase
OS: overall survival
RFS: relapse-free survival
STAT3: signal transducer and activator of transcription 3
TAMR: tamoxifen resistant
ZIP: Zrt, Irt-like protein
ZnT: zinc transporter protein

## Data Availability

Not applicable.
